# Mechanisms of Assembly and Cellular Interactions for the Bacterial Genotoxin CDT

**DOI:** 10.1371/journal.ppat.0010028

**Published:** 2005-11-18

**Authors:** Dragana Nesic, C. Erec Stebbins

**Affiliations:** Laboratory of Structural Microbiology, The Rockefeller University, New York, New York, United States of America; Yale University, United States of America

## Abstract

Many bacterial pathogens that cause different illnesses employ the cytolethal distending toxin (CDT) to induce host cell DNA damage, leading to cell cycle arrest or apoptosis. CDT is a tripartite holotoxin that consists of a DNase I family nuclease (CdtB) bound to two ricin-like lectin domains (CdtA and CdtC). Through the use of structure-based mutagenesis, biochemical and cellular toxicity assays, we have examined several key structural elements of the CdtA and CdtC subunits for their importance to toxin assembly, cell surface binding, and activity. CdtA and CdtC possess N- and C-terminal nonglobular polypeptides that extensively interact with each other and CdtB, and we have determined the contribution of each to toxin stability and activity. We have also functionally characterized two key binding elements of the holotoxin revealed from its crystal structure. One is an aromatic cluster in CdtA, and the other is a long and deep groove that is formed at the interface of CdtA and CdtC. We demonstrate that mutations of the aromatic patch or groove residues impair toxin binding to HeLa cells and that cell surface binding is tightly correlated with intoxication of cultured cells. These results establish several structure-based hypotheses for the assembly and function of this toxin family.

## Synopsis

The cytolethal distending toxin is used by many bacteria to damage the DNA of infected organisms. This DNA damage prevents cells from dividing and eventually leads to cell death, which raises the possibility that this genomic damage may be a contributing factor to carcinogenesis. The cytolethal distending toxin is composed of three proteins that form a tightly associated complex. After secretion by the bacterium, two proteins in this complex adhere to the cell surface and achieve the delivery of the third protein into the cell, where it causes DNA lesions. This report examines how this toxin is assembled and how it adheres to host cell surfaces. A set of molecular features on the toxin is shown to be critical for this cell adherence and for the ability of the cytolethal distending toxin to inhibit cell division. These results tie together for the first time aspects of the molecular structure of the cytolethal distending toxin and its ability to adhere to host cell surfaces, contributing to mechanistic understanding of the activity of this genotoxin.

## Introduction

The cytolethal distending toxin (CDT) is a tripartite bacterial toxin that targets many types of eukaryotic cells. Most of the cells intoxicated with CDT predominantly arrest in G2/M transition of the cell cycle; they slowly distend over a period of 2−5 d, and eventually die [1−4]. The effect on lymphocyte cultures is somewhat faster and different, since they do not distend but undergo apoptosis [5−7]. CDT activity has been detected among many pathogenic gram-negative bacteria that can cause a panoply of diseases such as chancroid, endocarditis, diarrhea, periodontal disease, and chronic hepatitis [1−4].

A major advancement in the understanding of the action of CDT was taken with the discovery that CdtB (cytolethal distending toxin B) shares weak active site sequence similarity with DNase I−like nucleases and that the mutation of predicted active site residues leads to a loss of CDT activity [8,9]. Subsequent studies demonstrated that CdtB has weak nuclease activity in vitro [10,11] and that CDT inflicts damage to eukaryotic DNA in vivo [4,5,9,10,12−15]. The crystal structure of the CDT holotoxin from *Haemophilus ducreyi* revealed that CdtB is indeed a DNase I−like nuclease in which all key residues involved in the catalysis and binding to DNA are conserved [16]. A growing amount of evidence supports a model of CDT intoxication by which CdtB delivery into the cells is mediated by CdtA (cytolethal distending toxin A) and CdtC (cytolethal distending toxin C) [17−19]. Once inside the cell, CdtB can translocate to the nucleus and induce DNA lesions, which will activate DNA damage response cascades and cause cell cycle arrest [18−20].

Very limited data are available about the function of CdtA and especially of CdtC. Only recently have several groups adapted different binding assays to study the interactions of CdtA and CdtC with eukaryotic cells. Most of these assays find that both CdtA and CdtC are capable of binding to the cellular membrane [21−24], and some suggested that they might share the same receptor [22,23]. Reports indicating that antibodies raised against CdtC were protective against CDT toxicity corroborated these findings [25]. On the contrary, Mao and DiRienzo [11] were not able to detect CdtC on the cell surfaces by using immunofluorescent techniques. The crystal structure of CDT holotoxin uncovered that both CdtA and CdtC are lectin-type structures, similar to each other and to the binding component of plant toxin ricin [16]. This realization, together with the positioning of lectin subunits, and the presence of two notable surface elements at their interface, an aromatic patch and an adjacent deep groove surface, suggest that the role of CdtA and CdtC is to interact with the cell surface and to enable translocation of the holotoxin across the plasma membrane [16].

In the present report, we have used structure-guided mutagenesis, biochemical and cellular assays, to examine the main structural features of CdtA and CdtC. We have discovered nonglobular interactions critical for the holotoxin assembly, stability, and cytotoxicity. Mutational analysis of the surface exposed residues of the aromatic patch and the groove shows that these two elements are critical to cell surface binding and toxicity.

## Results

### The Role of CdtA Nonglobular Interactions

We recently determined the crystal structure of *Haemophilus ducreyi* CDT [16]. The structure revealed that the toxin is composed of three different subunits (Figure 1A): CdtA and CdtC, which both exhibit a lectin-type fold, and CdtB, which is a DNase I-like nuclease. All three subunits interact intimately with each other, forming three extensive globular protein-protein interfaces. In addition, CdtA and CdtC each have extended, nonglobular polypeptides at their N- and C-termini, and in both proteins these regions interact with other elements of the holotoxin to cement the assembly of the ternary complex. These four nonglobular extensions, or “tails,” account for nearly a third of the surface area buried upon complex formation. These tails, therefore, likely contribute significantly to the stability of the ternary complex. By creating a series of deletion mutants, we have examined the role of the CdtA and CdtC “tails” in holotoxin assembly and activity.

**Figure 1:**
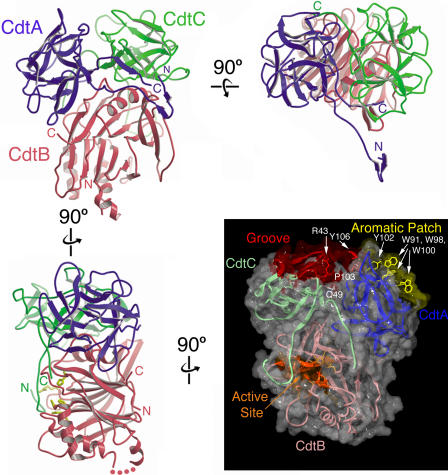
Structure of CDT and Mutational Locations Four alternative orientations of the crystal structure of the *H. ducreyi* CDT are shown as a ribbon cartoon tracing the three polypeptide chains. CdtA, CdtB, and CdtC are shown in blue, red, and green, respectively. Red dots indicate CdtB peptide not modeled due to disorder. N, NH_2_-terminus; C, COOH-terminus. The final image shows a partially transparent surface illustration focusing on the groove (red) and aromatic patch (yellow). Residues mutated for cell binding studies are indicated in white.

The N-terminus of CdtA is particularly interesting. Residues 18−56 are disordered and invisible in the crystal structure, and we sought to determine their contribution to the assembly and activity of the holotoxin. The amino acids 56−67 of CdtA make only minor contacts with other subunits within a single holotoxin complex, but instead interact with the groove of an adjacent holotoxin complex in the crystals [16]. The amino acids 67−75 of CdtA, however, do contact CdtB and CdtC in the appropriate complex in the crystal and therefore represent a more realistic structural interaction. Therefore, we created three N-terminal deletions of CdtA (Δ18−56, Δ18−67, Δ18−75) and examined them for complex assembly and toxin activity.

All mutants with N-terminal deletions of CdtA were successfully purified and refolded into CDT complex. The complex integrity in all cases was preserved during ion-exchange chromatography (Figure 2). The refolded complexes were loaded on a cation-exchange column (SP Sepharose Fast Flow; GE Healthcare, Piscataway, New Jersey, United States) at 40 mM NaCl concentration and eluted by gradual increase of salt concentration (from 0 mM to 500 mM NaCl). The CDT holotoxin, wild-type and all mutants, elutes between 100 mM and 150 mM NaCl. In addition, in some preparations even in the case of the wild-type complex, we observed a second peak eluting at concentrations higher than 200 mM NaCl. This peak was composed of the CdtB subunit only, the only CDT subunit that binds to SP Sepharose under these conditions (data not shown). That is not unexpected as the calculated pI of CdtB is fairly basic at 8.5, whereas CdtA and CdtC have acidic pIs of 5.58 and 6.21, respectively. It is possible that single peak of CdtB is due to excess CdtB protein in the refolding reactions that refolded apart from CdtA and CdtC, which then binds more strongly to the cation-exchange resin than the holotoxin with the acidic lectin domains. It is also possible that the cation-exchange column destabilizes the CDT holotoxin to some degree, leading to an increase in the “CdtB peak.” CdtA and CdtC were present in the flow through of the column as well, and this is what we observed with CDT holotoxin with Δ18−75 truncation of CdtA (Figure 2D). Moreover, when refolded in the absence of CdtB, CdtA and CdtC do not bind the resin under these conditions (data not shown).

**Figure 2:**
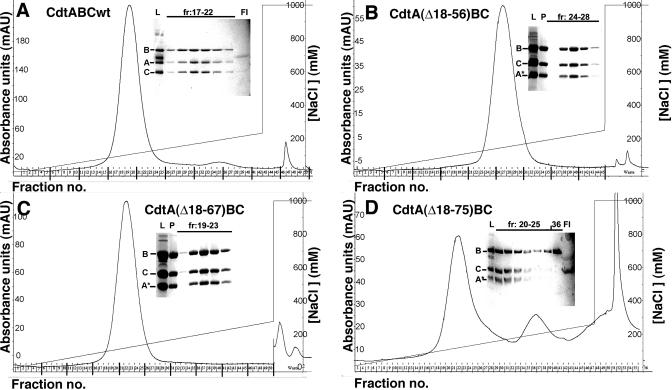
Cation-Exchange Chromatography of the CDT Holotoxin Containing N-Terminal Deletions of CdtA Wild-type (A) or mutant CDT holotoxins [(B) holotoxin with CdtA (Δ18–56), (C) holotoxin with CdtA (Δ18–67), (D) holotoxin with CdtA (Δ18–75)] were run on Fast Flow SP Sepharose columns using an ÄKTA FPLC. Refolded CDT complexes were loaded and examined in a salt gradient as described in Materials and Methods. Individual fractions (5 ml) or pooled material from the elution peak were collected and examined by SDS-PAGE. Proteins were stained with Coomassie blue stain. Images of the gels are presented inside corresponding chromatograms. L, loaded material; fr, fraction number; Fl, flowthrough; P, pooled material from elution peak. Absorbance was measured at 280 nm.

On a Superdex 200 gel filtration column (GE Healthcare), using a gel filtration buffer containing 200 mM NaCl, 20 mM HEPES (pH 7.5), 2.5 mM dithiothreitol (DTT) (Figure 3A), all CdtA deletion mutants that were refolded into a CDT holotoxin were shifted significantly to a lower molecular weight in gel filtration (Figure 3A). At the same time, all three subunits appear in a stoichiometric ratio in the peak fractions (Figure 3B). Moreover, all of these deletions of CdtA do not reduce the toxicity of the holotoxin, as measured by cell cycle arrest of holotoxin-treated cells (Figure 3C). One explanation for the discrepancy between the results for biochemical assembly and activity of the CdtA N-terminal deletion mutants would be to postulate an enhanced stabilization of the ternary complex upon interaction with a cell surface ligand. Indeed, gel filtration chromatography is a more demanding assay for complex stability than other chromatographic methods, and the three CdtA deletion mutants discussed above all co-purified by ion-exchange chromatography (Figure 2).

**Figure 3:**
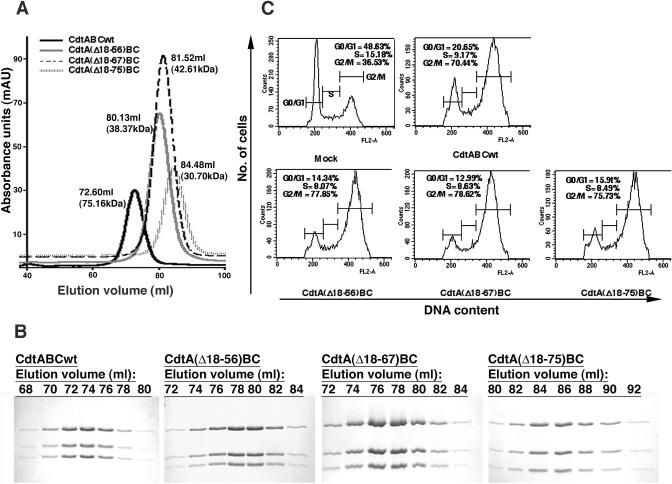
Effects of N-Terminal Deletions of CdtA on Holotoxin Integrity and Toxicity (A) Size exclusion chromatography profile of CDT holotoxin containing wild-type or deletion mutants of CdtA, wild-type CdtB, and *myc*-tagged CdtC. Following cation-exchange chromatography, 2-ml samples were injected onto a Superdex 200 HiLoad 16/60 column and run at flow rate of 2 ml/min in 200 mM NaCl, 20 mM HEPES (pH 7.5), and 2.5 mM DTT on an ÄKTA FPLC. Fractions of 2 ml were collected. Calculated molecular weights of globular proteins eluting at the same elution volumes are indicated in parentheses. (B) Peak fractions were analyzed by SDS-PAGE, and CDT subunits were visualized with Coomassie blue stain. A, CdtA; B, CdtB; C, CdtC; A*, deletion mutants of CdtA. (C) Cell cycle analysis of HeLa cells exposed to 1 ng/ml concentration of the wild-type or mutant holotoxin for 3 hr at 37 °C, 5% CO_2_. Cells were processed 48 hr after holotoxin treatment, and DNA content was measured by flow cytometry. The calculated percentages of cells in G0/G1, S, and G2/M are shown.

As an alternative to demonstrate complex formation, we have performed co-immunoprecipitation experiments. A Myc-tagged CdtC subunit was refolded together with wild-type CdtB and wild-type or deletion mutants of CdtA and subjected to ion-exchange and size exclusion chromatography. Gel filtration fractions were then subjected to immunoprecipitation with anti-*MYC* antibody. They were all able to co-immunoprecipitate, demonstrating that the subunits were still able to form complexes (Figure 4A) despite their disassembly during gel filtration chromatography. Nonspecific binding to protein G Sepharose was not detected (Figure 4B). All of these experiments suggest that contacts that the residues 18−75 of N-terminus CdtA make with other subunits contribute to the holotoxin stability, but they are not critical for complex formation and toxin activity.

**Figure 4:**
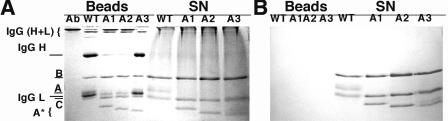
Immunoprecipitation of CDT Complexes Containing Deletion Mutants of CdtA (A) The complexes (10 μg each) were immunoprecipitated with 10 μg anti-myc antibody (myc tag was fused to C-terminus of CdtC) in 150 mM NaCl, 20 mM HEPES (pH 7.5), 0.1% Nonidet P40 substitute, followed by exposure to protein G Sepharose beads, and subjected to nondenaturing SDS-PAGE. (B) Nonspecific binding of CDT complexes to protein G Sepharose was not detected under the same experimental conditions. A, CdtA; B, CdtB; C, CdtC; A*, truncated CdtA; A1, holotoxin with CdtA Δ18−56; A2, holotoxin with CdtA Δ18−67; A3, holotoxin with CdtA Δ18−75; WT, wild-type; Ab, only anti-*myc* antibody, no CDT; IgG (H+L), nonreduced immunoglobulin G; IgG H, immunoglobulin G heavy chain; IgG L, immunoglobulin G light chain; SN, supernatant.

The C-terminus of CdtA extends to CdtC and binds by forming an intermolecular β-sheet, whereas the C-terminal extension of CdtC interacts with both CdtA and CdtB (Figure 1A). The CdtA C-terminus possesses a more extensive interaction than the N-terminal region of this subunit, and it might be expected that deletions of this region would have a more pronounced effect. This is indeed the case, as removal of this polypeptide (CdtAΔ215−223) leads to an inability of the complex to co-refold and to a precipitation of CdtA (data not shown).

### The Role of CdtC Nonglobular Interactions

We also made a series of deletion mutants of the CdtC N- and C- terminal tails, which demonstrate that the nonglobular interactions of CdtC with both CdtA and CdtB strongly contribute to toxin assembly, stability, and activity.

The N-terminal 13 amino acids of CdtC interact with active site residues of CdtB, and we have previously shown that these interactions play an autoinhibitory role in holotoxin activity in vitro [16]. We have previously shown that CdtCΔ(21−35) assembles into a biochemically stable holotoxin, and it is as active as the wild-type complex in toxicity assays [16]. A slightly larger deletion, CdtCΔ(21−39), which removes several residues that make a small number of additional contacts to CdtB and CdtA, destabilizes the complex. The mutant complex still co-purifies by cation-exchange chromatography, but there is a significant decrease in the holotoxin stability (Figure 6C). This is also evident by gel filtration chromatography (Figure 5A and 5B). This leads to impaired toxicity of the mutant holotoxin as determined by analysis of cell cycle of holotoxin-treated cells (Figure 5C).

**Figure 5:**
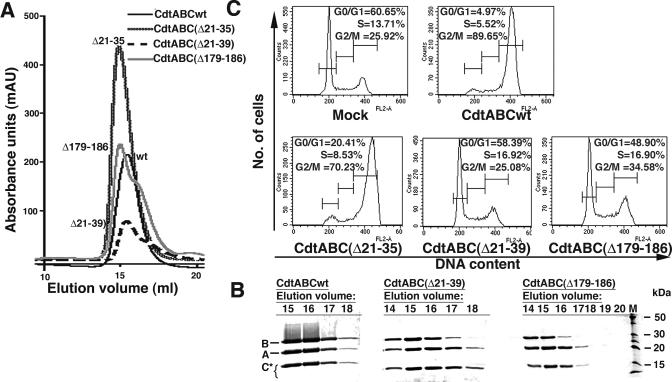
Effects of N- and C-Terminal Deletions of CdtC on Holotoxin Integrity and Toxicity (A) Size exclusion chromatography of recombinant holotoxin containing different CdtC mutants. After cation-exchange chromatography, 2-ml CDT holotoxin samples were injected onto a Superdex 200 HR 10/30 column and run at flow rate of 0.5 ml/min in 200 mM NaCl, 20 mM HEPES (pH 7.5), 2.5 mM DTT on an ÄKTA FPLC. Fractions of 1 ml were collected. (B) Following elution from the gel filtration column, several peak fractions were subjected to SDS-PAGE and CDT subunits visualized with Coomassie blue stain. A, CdtA; B, CdtB; C, cdtC; C*, deletion mutants of CdtC. (C) Cell cycle analysis of HeLa cells exposed to 1 ng/ml concentration of either wild-type or mutant holotoxin for 3 hr at 37 °C, 5% CO_2_. Cells were processed 48 hr after holotoxin treatment, and DNA content was measured by flow cytometry. The calculated percentages of cells in G0/G1, S, and G2/M are shown.

**Figure 6:**
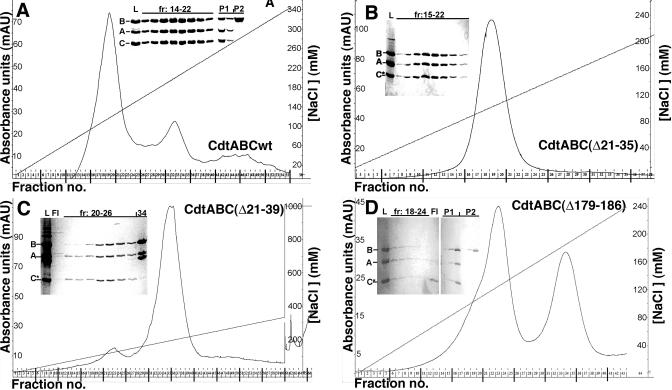
Cation-Exchange Chromatography of CDT Holotoxin Containing N- and C-Terminal Deletions of CdtC Wild-type (A) or mutant CDT holotoxins [(B) holotoxin with CdtC (Δ21−35), (C) holotoxin with CdtC (Δ21−39), (D) holotoxin with CdtC (Δ179−186)] were run on Fast Flow SP Sepharose columns (20 ml) using an ÄKTA FPLC. Refolded CDT complexes were loaded on an SP Sepharose column in buffer containing 40 mM NaCl, 20 mM HEPES (pH 7.5), 2.5 mM DTT and analyzed by a gradient in salt concentration as described in Materials and Methods. Individual fractions (5 ml) or pooled material (P) from elution peak were collected and examined by SDS-PAGE. Proteins were stained with Coomassie blue dye. Images of the gels are presented inside corresponding chromatograms. The peak that elutes between 100 and 150 mM NaCl concentration (P1) contains an intact CDT holotoxin, and the second peak (P2) that elutes at concentrations higher than 200 mM NaCl contains only the CdtB subunit. A, CdtA; B, CdtB; C, CdtC; C*, deletion mutants of CdtC; L, loaded material; fr, fraction number; Fl, flowthrough; P, pooled material from elution peak. Absorbance was measured at 280 nm. In A, P1 contains fractions 14−22, and P2 contains fractions 31−34; in D, P1 contains fractions 20−22, and P2 contains fractions 32−34.

As the C-terminal tail of CdtC interacts with both CdtA and CdtB, it is expected that its removal would undermine complex stability and decrease toxicity. CdtCΔ179−186, which deletes residues at the C-terminus that are not visible in the crystals, partially destabilizes the holotoxin assembly, as judged by gel filtration and ion-exchange chromatography (Figures 5A and 6D), and significantly reduces toxicity against cells (Figure 5C). It is not possible with these data to determine whether the decrease in toxicity is due to partial destabilization of the ternary complex or to a role of the CdtC C-terminus in receptor binding. A larger C-terminal deletion, CdtCΔ169−186, presents a much more forceful phenotype, as it does not productively refold, indicating that the loss of residues 169−178, which make contact with both CdtA and CdtB, more significantly destabilizes the assembly of the ternary complex (data not shown).

### Structural Basis of Cell Surface Binding

CdtA and CdtC have many similarities to the ricin B-chain in fold and in placement with respect to the active subunit within the holotoxin. Therefore, in analogy to ricin, it may be expected that these two subunits of CDT will have a function in host cell binding and internalization of the toxin. Several reports indicate that these subunits can adhere to cell surfaces [11,21−23]. The crystal structure shows that CdtA and CdtC together contribute toward the formation of two notable surfaces, a cluster of highly conserved aromatic residues, and a long and deep groove that is formed at the interface of two subunits (Figure 1B). These may represent regions responsible for cell contact and attachment. To address this possibility, we have altered these molecular surfaces by mutagenesis and examined mutant holotoxins for binding to cells and effectiveness to induce cell cycle arrest.

In order to be able to determine if different mutant holotoxins interact with the cells, we have developed a fluorescence-based binding assay. The holotoxin was fluorescently labeled with Alexa Fluor 488 fluorescent dye (Molecular Probes, Eugene, Oregon, United States). The Alexa Fluor 488 reactive dye has a tetrafluorophenyl ester moiety that reacts efficiently with primary amines of proteins to form stable dye-protein conjugates. It was necessary to test the activity of CDT-Alexa Fluor conjugate in a cellular intoxication assay, since there are reports suggesting that biotin labeling of CdtA disrupted complex formation and led to a reduction in toxicity [22]. Dye-conjugated CDT was as potent as unconjugated toxin and caused a comparable level of G2/M arrest of HeLa cells (Figure 7A). This enabled us to test the binding of fluorescently labeled holotoxin to HeLa cells by flow cytometry and confocal microscopy. Flow cytometry gave a quantitative evaluation of holotoxin binding, whereas confocal microscopy provided qualitative visualization of the cytometry results, while also revealing the localization of bound holotoxin. The labeled holotoxin specifically binds to cells in a manner easily detectable over background.

**Figure 7:**
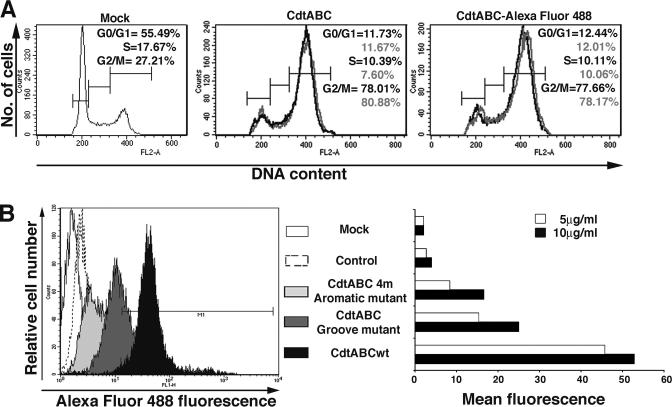
The Activity of CDT Holotoxin Labeled with Alexa Fluor 488 (A) The toxicity of CDT holotoxin labeled with Alexa Fluor 488. HeLa cells were treated with 1 ng/ml (black) or 10 ng/ml (gray) concentration of unconjugated or Alexa Fluor 488−conjugated CDT for 3 hr at 37°C, 5% CO_2_. Cells were processed 48 hr after holotoxin treatment, and DNA content was measured by flow cytometry. The calculated percentages of cells in G0/G1, S, and G2/M are shown. (B) Binding of CDT-Alexa Fluor 488 to cells. Harvested HeLa cells were exposed for 2 hr to 5 and 10 μg/ml concentration of wild-type or mutant CDT-Alexa Fluor 488. The histogram shows the binding of 5 or 10 μg/ml concentration of wild-type and mutant CDT-Alexa Fluor 488 conjugates to HeLa cells. Mock represents cells in buffer only (2% FCS in PBS), and control is goat anti-mouse IgG conjugated with Alexa Fluor 488, which does not bind to HeLa cells. The level of fluorescence was analyzed by flow cytometry. The relative levels of fluorescent labeling of wild-type and mutant CDT holotoxin was maintained to be nearly equivalent, with the mutant holotoxins (groove and aromatic patch) possessing a slightly higher level of labeling than the wild-type (Materials and Methods).

We have previously demonstrated that a quadruple aromatic patch mutant (W91G/W98G/W100G/Y102A) is incapable of intoxicating cells [16] (Figure 8B) and postulated that this prominent structural element could be involved in cell surface interactions. Using a fluorescence-based cell binding assay, we were able to show that this mutant is indeed incapable of binding to HeLa cells. By both flow cytometry and confocal microscopy, we were not able to detect any significant binding of the aromatic patch mutant above background (Figures 7B and 9). This shows that the aromatic patch residues are vital for cell surface attachment. A triple aromatic mutant (W98G/W100G/Y102A) behaves in the same way as the quadruple (Figure 8B).

**Figure 8:**
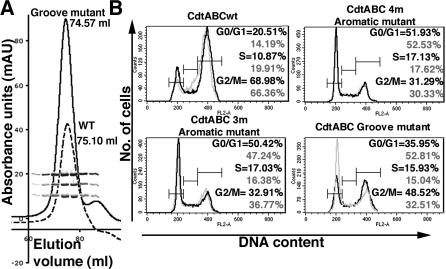
Effect of the Aromatic Patch and Groove Mutants on the Holotoxin Assembly and Toxicity (A) Elution profile of the wild-type and groove mutant of the CDT holotoxin during size exclusion chromatography. Samples were loaded on a Superdex 200 HiLoad 16/60 column at a flow rate of 2 ml/min in 200 mM NaCl, 20 mM HEPES (pH 7.5), 2.5 mM DTT on ÄKTA FPLC. Peak fractions (2 ml) were analyzed by SDS-PAGE (15% gel), and CDT subunits were visualized with Coomassie blue staining. SDS-PAGE of the CDT holotoxin groove mutant is shown inside gel filtration chromatogram. Elution volumes of wild-type and mutant holotoxin are shown. (B) Cell cycle analysis of HeLa cells exposed to 1 ng/ml (gray) or 10 ng/ml (black) concentration of either wild-type or mutant holotoxin for 3 hr at 37 °C, 5% CO_2_. Cells were processed 48 hr after holotoxin treatment, and DNA content was measured by flow cytometry, as detailed in Materials and Methods. The calculate percentages of cells in G0/G1, S, and G2/M are shown. CdtABC 4m Aromatic mutant (CdtA: W91G/W98G/W100G/Y102A); CdtABC 3m Aromatic mutant (CdtA: W98G/W100G/Y102A); CdtABC groove mutant (CdtA: P103A/Y106A, CdtC: R43K/Q49A).

**Figure 9:**
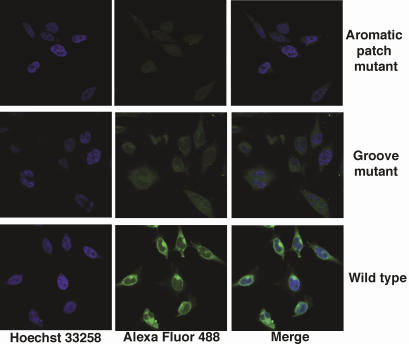
Binding of Mutant CDT- Alexa Fluor 488 Holotoxin to Cells HeLa cells were treated for 2 hr with 5 μg/ml concentration of wild-type or mutant fluorescently labeled holotoxin in 3% BSA/PBS at 37 °C, 5% CO_2_. Samples were co-stained with Hoechst 33258 dye to visualized nuclei. Toxin was washed out, and cells were fixed in 3.7% formaldehyde and mounted with ProLong Gold Antifade Reagent (Molecular Probes). Confocal images were taken under an inverted LSM510 confocal microscope (Zeiss) using ×60 objective lenses. The aromatic patch mutant contains wild-type CdtB and CdtC, and mutant CdtA (W91G/W98G/W100G/Y102A); the groove mutant contains wild-type CdtB and mutant CdtA (P103A/Y106A), and CdtC (R43K/Q49A).

We also mutagenized the extended groove formed by CdtA and CdtC to assess its importance. The groove mutant contains two mutations in CdtA (P103A and Y106A) and two in CdtC (R43K and Q49A). The rationale for these mutations was to include residues that are surface exposed and that also interact with the N-terminal tail of the symmetry-related CdtA molecule. The quadruple groove mutant did not affect complex stability as judged by ion-exchange (data not shown) and gel filtration chromatography (Figure 8A). However, the biological activity of the mutant holotoxin was significantly impaired (Figure 8B). We have examined the ability of the groove mutant to induce cell cycle arrest on HeLa cells using two different concentrations and found that the percentage of cells that arrest in G2/M is diminished at a 10 ng/ml concentration of toxin (compare wild-type [68.98%] and groove mutant [48.52%]), and it was almost undetectable at 1 ng/ml (the concentration that is usually sufficient to cause significant cell cycle arrest with the wild-type holotoxin). To characterize this mutant in more detail, we examined it in binding assays. Alexa Fluor 488−conjugated groove mutant exhibited noticeably diminished binding to HeLa cells as determined by both flow cytometry (Figure 7B) and confocal microscopy (Figure 9). It should be noted that the degree of labeling of the mutant CDT holotoxins was slightly higher than that of the wild-type CDT (see Materials and Methods), which excludes the possibility that lack of detection of binding was due to weaker fluorescent labeling. Therefore, these findings identify the groove as a second critical cell-contacting region of the holotoxin.

## Discussion

Lara-Tejero and Galan [18] initially proposed that CDT represents an “AB” type of toxin in which CdtB serves as the “A,” or active, subunit, and CdtA and CdtC represent a heterodimeric “B,” or binding, subunit. The crystal structure of the *H. ducreyi* CDT holotoxin strongly substantiated that hypothesis [16]. By analogy to other AB toxins [26], it has been proposed that CDT travels from the plasma membrane through an endocytic pathway and then enters the Golgi network and endoplasmic reticulum, after which CdtB translocates directly into the nucleus [27], or is delivered by retrograde transport into the cytosol, and then translocates into the nucleus [17−20]. In order to try to elucidate the role of CdtA and CdtC in the toxin assembly and cellular binding, we have addressed the contribution of several critical structural elements of these molecules by biochemical and cellular assays.

Using deletion mutagenesis, we examined the role of several nonglobular interactions in toxin stability and function. We found that contacts of C-terminal tails of CdtA and CdtC with other subunits are vital for toxin assembly. Their removal results in the poor refolding of the holotoxin, destabilization, and, as a consequence, inefficient cellular intoxication. The N-terminal tail of CdtC is deeply inserted into the active site of CdtB, which may contribute to overall stability of the complex. Interestingly, the truncation of 35 amino acids from the N-terminus of CdtC does not disturb complex stability, but removal of just four more residues leads to destabilization of the holotoxin and impaired toxicity. On the other hand, the N-terminal tail of CdtA makes only a few contacts with other subunits, which appear to be inconsequential for the CdtA function in the holotoxin. Although removal of up to 75 residues from N-terminal tail of CdtA destabilizes the complex under gel filtration conditions, truncated mutants are still as potent as the wild-type holotoxin against target cells. This finding is in agreement with previous observations that deletion 19−49 of closely related *Actinobacillus actinomycetemcomitans* CdtA [28] or, in another report, the first 59 residues [29] are dispensable for holotoxin activity. Moreover, several groups have demonstrated that some preparations of holotoxin actually contain truncated CdtA [25,30]. In other bacterial pathogens, the region N-terminal to ricin fold of CdtA is very diverse and much longer than in *H. ducreyi*. It is possible that these long N-terminal tails play a role in the assembly and translocation of the holotoxin in bacterial cells.

Recently, several studies examined the binding of CDT subunits to target cells and addressed the role of both CdtA and CdtC. The results were somewhat conflicting in the conclusion of what contribution CdtC made to this process. While several groups have shown that both CdtA and CdtC can bind to the surface of HeLa cells and that preincubation with CdtA-CdtC complex can inhibit subsequent intoxication with holotoxin [21−24], others have observed only binding of CdtA to Chinese hamster ovary cells [11]. Furthermore, it was suggested that CdtA and CdtC might bind to the same cellular receptor [22,23]. The crystal structure of *H. ducreyi* CDT holotoxin demonstrated that both CdtA and CdtC are lectin-type structures, and they both contribute to the formation of two prominent molecular surfaces that could be involved in cellular interactions. The first is a region composed of highly conserved, surface-exposed aromatic residues. We have previously shown that mutations of four CdtA aromatic residues (W91G, W98G, W100G, Y102A) abolish the holotoxin activity [16]. Here, using fluorescence-based binding experiments, we were able to demonstrate that these residues are indeed involved in the cell surface binding. While binding of fluorescently labeled wild-type holotoxin to HeLa cells was readily detectable by flow cytometry and confocal microscopy, binding of an Alexa Fluor 488−conjugated aromatic mutant was undetectable in both assays. It appears that the disruption of only three aromatic residues of CdtA (W98, W100, Y102) is sufficient to abolish cellular interactions and abrogate holotoxin activity. It was recently shown that *Escherichia coli* CdtA-II and CdtC-II can interact with glycoproteins, and it was postulated that the putative cellular receptor would most probably have an N-linked fucose-containing structure [23]. It has been suggested that the aromatic patch could be a potential carbohydrate-interacting region [23,31], although the lipophilic nature of this cluster of aromatic residues suggests that it may also serve to interact with components of the plasma membrane. Indeed, it has been recently shown that interaction of CDT with the cells is sensitive to cholesterol depletion, and it was therefore suggested that cholesterol-rich lipid rafts could be involved in CDT binding [27].

We have also identified an additional cell-binding region on the surface of the CDT holotoxin. It is represented by a groove that is formed by CdtA and CdtC along their molecular surfaces. Mutations of four surface-exposed groove residues (two in CdtA:P103A/Y106A and two in CdtC:R43K/Q49A) significantly decrease binding to HeLa cells, which results in the decrease in CDT toxicity. The effect of these mutations was less severe than that of mutations of aromatic residues, representing an intermediate phenotype. By increasing concentrations of the groove mutant, it was possible to induce more robust cell cycle arrest. In contrast, aromatic mutants were completely incapable of causing any effect on treated cells. This could suggest that these two regions are involved in two independent binding events with the cell surface.

Our findings are an important step toward a better understanding of the molecular mechanisms underlying CDT holotoxin assembly and interaction with the target cell surfaces. Understanding toxin assembly and activity also provides potential targets for pharmacological disruption of CDT activity. Finally, the identification of host cell surface binding mutants of CDT provides important tools for the identification of host cell receptors mediating toxin entry.

## Materials and Methods

### Production of wild-type and mutant *H. ducreyi* holotoxin.

*H. ducreyi* CDT subunits were cloned, purified, and holotoxin complex reconstituted as previously described [16]. CdtA(18−223), CdtB(23−283), and CdtC(21−186) were cloned by PCR from genomic DNA (ATCC [American Type Culture Collection] number 700724D, Manassas, Virginia, United States) as an N-terminal hexahistidine fusion proteins in *E. coli* such that the predicted N-terminal secretion signals were removed [32]. *cdtA* was cloned in pET-28a vector (Novagen, Madison, Wisconsin, United States) with engineered in-frame rhinovirus 3C protease recognition sequence at the 5′ end of *cdtA*. *cdtB* was cloned into an engineered version of the pac28 vector [33], which also contains a 3C protease recognition sequence between the hexahistidine tag and the *cdtB*. *cdtC* was cloned in the pET-21a vector (Novagen), which has an insertion with a hexahistidine tag and an engineered 3C protease recognition site. The three subunits were expressed separately in BL-21 *E. coli* cells by the addition of 0.1 mM isopropyl-β-D-thiogalactopyranoside (IPTG; Gold Biotechnology, Inc., St. Louis, Missouri, United States) for 4 hr at 37 °C, 200 rpm. The subunits were purified under denaturing conditions (8 M urea, 10 mM Tris [pH 8.0], 0.1 M Na-phosphate) using nickel chelating affinity resin (GE Healthcare). Each subunit was eluted from nickel chelating affinity resin with the elution buffer that contained 500 mM imidazole (pH 8.0), 8 M urea, 10 mM Tris (pH 8.0), 0.1 M Na-phosphate. The usual yields per liter of bacterial culture were 50 mg of CdtA, 60 mg of CdtB, and 200 mg of CdtC. The CDT holotoxin was reconstituted by co-refolding all three subunits together via dialysis at 4°C into a native buffer consisting of 20 mM HEPES (pH 7.5), 200 mM NaCl, 2.5 mM DTT, 5% glycerol, and 2 mM EDTA. The total protein concentration during refolding was maintained under 0.1 mg/ml (20 mg of protein in 200 ml of denaturing buffer per 4 L of native buffer), and the native buffer was changed four times within a 24-hr period. The proteins were separated from the affinity tag through site-specific proteolytic cleavage with rhinovirus 3C protease (2 μg/ml 3C protease fused to a GST tag) for 12 hr at 4 °C. The protease was subsequently removed by passing the material through a Fast Flow GST-Sepharose column (GE Healthcare). The CDT holotoxin was further purified by cation-exchange chromatography (SP Sepharose Fast Flow, 20 ml bead volume; GE Healthcare) using an ÄKTA FPLC (GE Healthcare). Refolded material was diluted five times to lower the salt concentration to 40 mM NaCl prior to loading the SP Sepharose column. After equilibrating the column with washing buffer (20 mM HEPES [pH 7.5], 2.5 mM DTT), the column was subjected to a gradient increase in salt concentration (from 0 to 500 mM NaCl in 20 mM HEPES [pH 7.5], 2.5 mM DTT during 150 min at flow rate of 3 ml/min). The remaining material was bumped from the column with 1 M NaCl, 20 mM HEPES (pH 7.5), 2.5 mM DTT. Individual fractions (5 ml) or pooled material from elution peak was collected and examined by SDS-PAGE. As a final step in the purification, and at the same time as a test of complex integrity, fractions eluted from the SP Sepharose column that contained intact CDT holotoxin were pooled together, concentrated using Centricon Plus-20 (Millipore, Billerica, Massachusetts, United States), and run on a gel filtration column (Superdex 200 HiLoad 16/60 [120 ml] or Superdex 200 HR 10/30 [25 ml]; GE Health), as indicated in the figure legends. Gel filtration buffers contained 200 mM NaCl, 20 mM HEPES (pH 7.5), 2.5 mM DTT; a 2-ml sample was injected, and 2-ml (from Superdex 200 HiLoad 16/60) or 1-ml (from Superdex 200 HR 10/30) fractions were collected. The peak fractions were examined by SDS-PAGE, and proteins were visualized by staining with Coomassie blue staining solution (50% methanol, 0.05% Coomassie brilliant blue R-50, 10% acetic acid in water) followed by destaining solution (5% methanol, 7% acetic acid in water).

The deletion mutants were generated by PCR and inserted into the same vector as the wild-type genes. The amino acid substitutions were introduced into *cdt* genes by PCR using *Pfu* DNA Polymerase (Stratagene, La Jolla, California, United States) and primers containing the appropriate base changes. The template plasmid was subsequently removed by digestion with DpnI prior to transformation. Myc tag sequence (EQKLISEEDL) was fused in-frame to the 3′ end of the wild-type *cdtC* gene in two subsequent PCRs using the pET-21a-CdtC plasmid as template, a T7 promoter primer as a forward primer in both reactions, and myc1R (5′-GAGTTTCTGCTCGCTACCCTGATTTCTTCG-3′) and myc2R (5′-TTACAGATCCTCTTCAGAGATGAGTTTCTGCTCGCTACC-3′) as reverse primers. The PCR product was initially cloned into the pCR2.1 TOPO vector (Invitrogen, Carlsbad, California, United States), and after sequence verification subcloned into the pET21a. Purification of the mutant proteins and assembly into the mutant CDT holotoxin were performed as previously described for the wild-type complex.

### Cell cycle analysis/cellular intoxication assay.

As a measure of toxin potency and cellular intoxication, we have examined by flow cytometry the cell cycle progression of HeLa cells treated with the CDT holotoxin. HeLa cells (2 × 10^6^ cells) were treated with indicated concentrations of wild-type or mutant CDT holotoxin for 3 hr at 37 °C, 5% CO_2_, and were subsequently washed of toxin and maintained in culture media. At 48 hr after treatment with CDT, cells were harvested, fixed by the addition of cold 70% ethanol during continuous vortexing, and left at 4 °C for 16 hr. The cells were then stained for 2 hr at 23 °C with propidium iodide solution (25 μg/ml propidium iodide, 100 U/ml DNase-free RNase A, 0.04% Triton X-100 in PBS). Relative DNA content was measured by flow cytometry with an FACSCalibur flow cytometer (Becton-Dickinson, Palo Alto, California, United States). The data were collected from 20,000 cells, and analysis was performed with Cell Quest software.

### Labeling of the CDT holotoxin with Alexa Fluor 488 fluorescent dye and flow cytometry.

The CDT holotoxin was conjugated with Alexa Fluor 488 dye using the Alexa Fluor 488 Monoclonal Antibody Labeling Kit (Molecular Probes) according to the manufacturer's instructions. Briefly, 100 μg of CDT holotoxin in PBS/0.1 M sodium bicarbonate (1 mg/ml final protein concentration) was added to the vial of reactive dye and incubated overnight at 4 °C on a rotator shaker. Labeled protein complex is then separated from unconjugated dye using a purification resin supplied by manufacturer. The degree of labeling was determined by measuring the absorbance at 280 nm and 494 nm using a NanoDrop ND-1000 spectrophotometer (Wilmington, Delaware, United States) and calculating the protein concentration and the fluorescent dye concentration using Protein & Labels module of ND-1000 software. The degree of labeling was calculated as moles of dye per mole of protein. This produced calculations of labeling of 2.98 ± 0.11 for wild-type CdtABC, 3.99 ± 0.32 for groove mutant CdtABC, and 5.64 ± 0.33 for aromatic mutant. Goat anti-mouse IgG was purchased already conjugated with Alexa 488 (Molecular Probes), and the degree of labeling was 6.4 moles of dye per mole of protein.

Binding of fluorescently labeled CDT holotoxin to HeLa cells was determined by flow cytometry. HeLa cells (1 × 10^6^) were harvested, washed twice with PBS, and incubated for 2 hr at room temperature with indicated amounts of wild-type or mutant CDT holotoxin in 2% FCS/PBS. Goat anti-mouse IgG antibody conjugated with Alexa Fluor 488 (Molecular Probes) was used as a negative control. Samples were washed three times with PBS and fixed with 1% formaldehyde (Polysciences, Inc., Warrington, Pennsylvania, United States) in PBS prior analysis. The presence of green fluorescence on HeLa cells was measured by flow cytometry using an FACSCalibur flow cytometer (Becton-Dickinson). Analysis was performed with Cell Quest software.

### Immunoprecipitation of CDT complexes.

CDT complexes composed of either mutant or wild-type CdtA, wild-type CdtB, and CdtC fused in-frame with *myc* tag at the C-terminus were immunoprecipitated using an anti-*myc* antibody (9E10; Santa Cruz Biotechnology, Santa Cruz, California, United States). Then, 10 μg of protein complex was incubated with 10 μg of antibody in binding buffer (20 mM HEPES [pH 7.5], 150 mM NaCl, 0.1% Nonidet P40 Substitute [Sigma, St. Louis, Missouri, United States]) for 3 hr at 4 °C. Immune complexes were incubated with protein G Sepharose 4 Fast Flow (Molecular Probes) in the binding buffer for additional 3 hr at 4 °C. Immunoprecipitates were collected by centrifugation, washed five times with binding buffer, and resuspended in nonreducing sample buffer (2% SDS, 50 mM Tris-Cl [pH 6.8], 0.1% bromophenol blue, 10% glycerol). The samples were boiled for 3 min at 95 °C, briefly centrifuged, and subjected to SDS-PAGE. Protein gels were stained with Coomassie blue dye. Nonreducing conditions were used to minimize dissociation of heavy and light immunoglobulin chains, which masked CDT subunits on a Coomassie blue−stained gel. It was not possible to avoid complete separation of the immunoglobulin chains due to the fact that CDT complexes were already in the buffer with DTT.

### Confocal microscopy.

HeLa cells, cultured directly on microscope cover glass, were treated with 5 μg/ml concentration of mutant or wild-type CDT holotoxin labeled with Alexa Fluor 488 in PBS buffer containing 3% BSA and Hoechst 33258 dye (1:500) (Sigma). Following treatment, cells were washed three times with PBS and fixed by incubation in 3.7% formaldehyde for 15 min at room temperature. Fixative was replaced with PBS, and samples were mounted with ProLong Gold Antifade Reagent (Molecular Probes). Samples were dried for at least 48 hr prior to examination under inverted LSM510 confocal microscope (inverted Zeiss Axiovert 200 microscope, Thornwood, New York, United States) using ×60 objective lenses. Images were collected and analyzed using LSM510 software (Zeiss).

## Supporting Information

### Accession Numbers

The GenBank (http://www.ncbi.nlm.nih.gov/Genbank) accession number for *Haemophilus ducreyi* CDT is U53215. The Protein Data Bank (http://www.rcsb.org/pdb) accession number for the crystal structure of *H. ducreyi* CDT is 1SR4 and is shown in Figure 1.

## Abbreviations

CDT: cytolethal distending toxin
DTT: dithiothreitol
