# Correction: Mechanisms of Assembly and Cellular Interactions for the Bacterial Genotoxin CDT

**DOI:** 10.1371/annotation/d5831b82-910f-4acc-82e3-ee0e006fd6bf

**Published:** 2008-10-14

**Authors:** Dragana Nesic, C. Erec Stebbins

The acknowledgments should have contained a funding reference to U54AI057158 (Northeast Biodefense Center-Lipkin).

